# HEALTHY AGING TALK AROUNDS: NEAR AND FAR

**DOI:** 10.1093/geroni/igac059.2583

**Published:** 2022-12-20

**Authors:** Lindsay Mullins

**Affiliations:** Franciscan Missionaries of Our Lady University, Baton Rouge, Louisiana, United States

## Abstract

BackgroundLoneliness and chronic health conditions are the two most cited ailments among older adults (OAs) that deter independent living. Community-tailored health programs increase feelings of connectivity to neighbors and link OAs to healthcare resources. MethodsDesign. The Healthy Aging Talk Around program is product of CAB formation, input, and a series of talks in-person (near) and virtually (far). Using qualitative methods, data was collected to determine significant health issues for OAs and qualitative data were collected through surveys to evaluate knowledge, access, and feelings of connectivity related to series gatherings. Setting and Population. Inner-city and rural OAs in the deep south from various communities (2 FBOs, 1 Assisted Living, 2 neighborhood groups). Series was held in community and later online. Attendance ranged between 75-130 OAs. Measures. Qualitative measures included pre and post series surveys (n=110; n = 91) with questions to determine the impact of the series. Analysis. Content analysis was conducted with the qualitative data to determine the impact of the series on 1.) knowledge of health issue(s), 2.) likelihood of accessing health resources, and 3.) feelings of connectivity and isolation. ResultsThe qualitative data analysis suggested the series as relatable because information was tailored linked to local community resources. Discussion of independent living challenges fostered connectivity with neighbors. Once virtual, knowledge and access were similar, feelings of connectivity were less prevalent. ConclusionA community-tailored health program series can increase knowledge of health issues, access to resources, and create neighborly connectivity potentially influencing ability to age in place.

